# Worker perspectives on the impact of non-standard workdays on worker and family well-being: A qualitative study

**DOI:** 10.1186/s12889-021-12265-8

**Published:** 2021-12-08

**Authors:** Adekemi O. Suleiman, Ragan E. Decker, Jennifer L. Garza, Rick A. Laguerre, Alicia G. Dugan, Jennifer M. Cavallari

**Affiliations:** 1 Department of Public Health Sciences, UConn School of Medicine, 263 Farmington Ave MC6325, Farmington, CT 06030-6325 USA; 2 Department of Psychological Sciences, Division of Industrial and Organizational Psychology, University of Connecticut, Storrs, CT 06269 USA; 3 Department of Medicine, Division of Occupational and Environmental Medicine, UConn School of Medicine, Farmington, CT 06030 USA

**Keywords:** Shift work, Long hours, Worker well-being, Work schedule

## Abstract

**Background:**

Non-standard work schedules (NSWSs), occurring outside of regular and predictable daytime hours, may negatively affect worker and family health. This qualitative study sought to understand worker perspectives on the health and well-being impacts of NSWSs among full-time, transportation maintainers, correctional, and manufacturing workers.

**Methods:**

Forty-nine workers participated in 8 focus groups. Data were transcribed and analyzed with ATLAS.ti, using the constant comparative method to identify themes and sub-themes.

**Results:**

Workers reported that long work hours and irregular and unpredictable schedules posed the biggest obstacles to their well-being. Workers reported that NSWSs were associated with behavior impacts (poor family and social connections, poor eating, poor sleep, lack of exercise recovery), physical health impacts (exhaustion, weight gain) and extended work exposures (increased stress, increased accidents).

**Conclusions:**

This highlights the importance of developing and implementing effective workplace interventions to address these barriers to health and health behaviors.

## Background

Non-standard work schedules (NSWSs), characterized by work schedules occurring outside of the traditional 9 AM to 5 PM workday, Monday through Friday pattern are common and increasing in the US workforce. While NSWSs have long been commonplace for workers in some sectors such as public safety (e.g. police), healthcare, and retail, the expansion of NSWSs to a broader range of work sectors is new. Historic estimates suggests that 90% of Americans have worked a NSWS at some point in their life [1, 2], although given recent disruptions in work schedules due to the COVID-19 pandemic, the prevalence of NSWSs is likely even higher.

In addition to being characterized as standard or non-standard, time at work can be described through a number of characteristics. “Shift work,” is one type of NSWS, defined as work scheduled outside the typical daytime work period that may extend beyond a typical 8 to 9 hour schedule, in order to meet the staffing needs of organizations that operate continuously over 24 hours [3]. It may include on-call work, compressed work schedules, rotating shifts, weekend work and many other shift arrangements. One comprehensive framework put forth by Harma and colleagues [4] characterizes work based on seven domains that include (1) shift length, (2) time of day, (3) shift intensity, (4) schedule control, (5) schedule predictability, (6) free time, and (7) variability of working time. While NSWSs help to meet the societal and economic demands of a 24/7 society, there are implications for workplaces, workers, as well as families and communities [5].

It is hypothesized that NSWSs, and shift work more specifically, impact workers through three mechanisms: circadian rhythm disruption, fatigue, and social rhythm disruption [6]. Circadian rhythm disruption occurs when a worker’s sleep schedule is outside of the natural day/night, wake/sleep pattern. With continued disruption, circadian rhythm misalignment can lead to cell proliferation, increased inflammation, and immunosuppression, putting workers at increased risk for breast cancer, cardiovascular disease, gastrointestinal and metabolic disorders, and possibly other cancers, mental health and reproductive problems [6]. Circadian rhythm disruption can occur when workers are working in the late evening and early morning hours as can be the case with shift work.

Fatigue occurs when there is insufficient time for recovery and sleep, which increases the risk of injuries and accidents [7] and may lead to a cascade of inflammatory responses implicating numerous chronic diseases including metabolic disorders, cardiovascular disease, cancer and diabetes [6]. Long work hours, including long work weeks (>55 hours worked per week), as well as shift work can all disrupt sleep patterns and lead to fatigue.

Social disruption occurs when workers are unable to participate in the stable social rhythm of evening and weekend activities that are consistent with western industrialized societies [8]. In addition to impairing social life, social disruptions can precipitate work-life conflict [8] and may also play a role in the link between poor work schedule characteristics and depression [9]. Working in the evening or the early morning hours, work on the weekend, long work hours, and irregular and unpredictable work hours have all been linked to social disruptions [8]. NSWSs have also been linked to reduced family well-being including increased risk of separation or divorce and poorer emotional and developmental outcomes for children whose parent works shift-work [5, 8].

The mechanisms by which working hours impact worker and family well-being help to inform our understanding of the harmful characteristics of work schedules [10, 11]. However, the characteristics of work schedules and the mechanisms by which they have effects tend to overlap. Long work hours can extend into the evening and early morning hours with fatigue and circadian disruption simultaneously affecting worker well-being while producing stressors on personal life adding social disruption.

Previous occupational research examining the effects of work schedules on worker and family well-being have primarily relied on quantitative methods. However, a qualitative research approach, driven by participants’ interpretation of their environment and assessment of their well-being allows researchers to gain an in-depth understanding of complex relationships and has been successfully applied among other workers [12–14].

The purpose of this study was to better understand the range and depth of well-being effects of work schedules in workers who experience a combination of both long and irregular work hours. The study cohort included blue-collar workers from a variety of industry sectors including transportation, corrections, and manufacturing. Workers from these populations were employed full-time with access to benefits, yet had varied exposures to working time characteristics. These blue-collar workers were also similar in their financial situation and geographical location. In addition to exposures to NSWSs, blue-collar workers are often times also exposed to additional hazardous work organization exposures such as monotonous and repetitive work and low levels of work control [15].

## Methods

### Study design

This was a qualitative study that used focus groups to assess worker perceptions on how time at work impacts worker and family well-being. It was the first phase of the larger mixed-methods, cross-sectional, WorkTime study, based on a participatory research approach involving workers from three sectors: transportation, corrections, and manufacturing. Focus groups (*n*=8) were performed with a subset of workers from these sectors to understand how their experience of NSWSs impacts the health and well-being of themselves and their families. The study was approved by the UConn Health's Institutional Review Board and written informed consent was received from all research participants.

### Focus group participants and recruitment

We provided work sites with recruitment flyers, briefly describing the study and purpose of the focus group that were handed out to potential participants ahead of time. All workers who were willing to share their NSWS experiences were recruited through convenience sampling at their work site. Within the transportation sector, we recruited transportation maintainers or “maintainers” who perform maintenance and repair of roadways. Within the corrections sector, we recruited both correctional officers and supervisors. We recruited manufacturing workers from a privately owned, small manufacturing company. We continued to recruit participants until no new themes emerged i.e., data saturation was reached across a broad range of NSWS experiences across industries. Focus groups included representatives from only one industry at a time.

### Focus group script

A semi-structured focus group script was designed by the research team to understand work hours and schedule concerns at different work sites, characterize how work hours and schedules acted as barriers to worker and family well-being, and consider ways in which work hours or schedules could be beneficial to worker well-being (Table 1). The script was pilot tested and refined before use in the field. The research team included experts in occupational health, industrial hygiene, occupational health psychology and industrial and organizational psychology. The focus group script was guided by the framework of NSWSs put forth by Harma et al. [4], the mechanisms by which NSWSs effect worker and family well-being [8], as well as initial conversations with workers who experience NSWSs. Consistent with the U.S. National Institute for Occupational Safety and Health (NIOSH) *Total Worker Health®* approach [2], we took a broad view of well-being which in addition to being associated with good health, included health behaviors, mental health, and social connections. This concept of well-being was described to the workers during the focus group.

**Table 1:** Question guide for focus groups

Primary question	Possible probes
**Health and work hours/schedule**	
Let’s talk about your work hours and schedule.	
1. Briefly describe the type of work that you do.	
2. What is your typical work hours and work schedule?	a. How long is your shift? b. What are your work start and end times? c. What days do you work over a week? Month? Season?
3. How do your work hours and/or schedule change?	a. How does your work start and end times and/or shift length change? b. How do the days you work over a week change? Month? Season? i. What causes your work hours or schedule to change? Weather? Production changes? Staff shortage or absences? ii. How often are your work hours and/or schedule changed? iii. How much advance notice of work hours/shift changes are you given? iv. Are you ever on-call? For how long? v. Why do you work extra work hours/shifts? vi. Are the extra work hours/shifts mandatory or voluntary? c. Outside of this job, what other types of work (paid or unpaid) do you do?
4. What aspects of your work hours and schedule are the biggest obstacle to your health and well-being?	a. How does your work hours/schedule: a. Increase your stress at work? Outside work (i.e., at home, with family)? b. Get in the way of your ability to: i. Sleep or relax? ii. Participate in healthy activities? iii. Participate in leisure activities? iv. Be involved in home or family activities? v. Be involved in community activities? vi. Have good relationships with family or friends?
5. Are there ways that your work hours or schedule benefit your health or well-being (being happy and enjoying life)?	

### Focus group procedure

We conducted the focus groups at the worksites, with only the research team (consisting of the facilitator and one research assistant) and the participants in attendance. Participants took a brief demographic survey, administered electronically, before the focus groups commenced. Focus groups were audio recorded with the knowledge and permission of the participants, while the research team took detailed notes to provide context of non-verbal expressions. Participants were asked to use only first names to assure confidentiality. Eight focus groups were conducted separately among a total of 49 workers (25 from transportation, 17 from corrections, and 7 from the manufacturing company). Each focus group had on average 6 participants with focus group size ranging from 4 to 8. A member of the research team facilitated the focus groups using the focus group script with question guides, and probes were used where appropriate (Table 1). Each focus group lasted about 1 hour (median 35 minutes) and were conducted over a 4-month period (April 2018 to August 2018).

### Data analysis

We computed descriptive summary statistics of demographic data, including means, standard deviations, and frequencies using SPSS version 25. The audio-recording from the focus groups were professionally transcribed and reviewed for accuracy. Transcripts were imported into Atlas.ti version 8 qualitative software package for analysis. Analysis of the transcribed data was done using the constant comparative method of qualitative data analysis [16] to generate meaningful concepts and theories through identifying common themes and sub-themes which were quantified across all focus groups, until no new themes emerged. Data from each of the eight focus groups was coded, and then reviewed as a combined dataset.

Coding used the inductive approach, which was driven by the available data [17]. One of the members of the research team completed the initial coding by reading through the transcripts line by line, to identify significant words or phrases (quotations) to generate codes. Sub-themes and themes were subsequently identified from the emerging codes and codes groups by grouping common quotations and codes into codes and code groups, respectively. The team continually discussed and reflected on the emerging sub-themes and themes to ensure validity. A final code structure evolved based on continuous review by two of the researchers, and also from input from worker teams consisting of a subset of the initial focus group participants. Based on the final sub-themes and themes, two members of the research team independently re-coded the data, and calculated the inter-coder agreement (ICA), which was at 87 percent agreement. A third member of the team served as the tiebreaker in cases where there were code discrepancies. Sub-theme frequencies were derived from code densities.

When workers were asked about the aspects of their work hours and schedules that were the biggest obstacles to their health and well-being, 9 sub-themes emerged, leading to 3 main themes: well-being behavior impacts, physical health impacts and extended exposures from work. 

## Results

Forty-nine workers participated in the focus groups (Table 2). The participants on average were 46 years old (SD = 10). The majority of workers were male (92%), White (51%), with more than a high school diploma or its equivalent (51%), married (70%), and had either a child or adult dependent (81%). Most of the participants were transportation maintainers (51%), worked the first shift (84%), and had no supervisory responsibilities at work (71%).

**Table 2:** Characteristics of focus group participants (*n* = 49)

Characteristic	N(%) or Mean (SD)
Age (years)	46 (10)
Male	45 (92)
Race	
African, African American, Black European Descent, White Multiracial and other races	12 (25) 26 (53) 11 (22)
Education	
Less than high school diploma or equivalency High school diploma or equivalency Associate’s degree, certificate, or some college Bachelor’s degree Master’s degree	1 (2) 23 (47) 17 (35) 6 (12) 2 (4)
Marital status	
Married or live with partner Divorced or separated Single, never married	34 (70) 7 (14) 8 (16)
Dependents	
Adult dependents Child dependents	15 (32) 24 (49)
Industry	
Transportation Corrections Manufacturing	25 (51) 17 (35) 7 (14)
Shift	
First shift Second shift Irregular schedule or hours Multiple	41 (84) 1 (2) 1 (2) 6 (12)
Supervisory responsibility	
No supervisory responsibility Team leader, supervisor, or manager	35 (71) 14 (29)

Workers were asked to describe their work, typical work hours and schedules, and how their work hours and schedules change (Table 1). Maintainers reported that their job involved highway/road maintenance - patching potholes, paving, repairing guardrails, mowing grass, flagging, brush cutting, picking-up litter, masonry and wood chipping in the summer and snow plowing in the winter. They described working long, irregular and unpredictable schedules mostly during winter storms, typically about seventeen to twenty-one hours per day, with short breaks. Schedules also often changed with short notice and calls-in during the winter. A maintainer described his work hours below:

*“So after the 14*^*th*^ *hour, if you rested three hours and say we were staying, you would go another 17 hours. Your second rest break would be four hours, which we’ve only done maybe once or twice this whole winter. We didn’t have too many four-hour rest breaks this year.”* (Male transportation maintainer)

Correctional officers (CO) reported that their work involved monitoring inmates’ daily activity inside the correctional facility to ensure their safety, health and welfare. They work different eight-hour shifts - first, second and third, often with rotating schedules and a lot of voluntary and mandatory overtime. Correctional supervisors were assigned to supervise the different shifts and were responsible for many administrative duties. They also work long, unpredictable hours with changing schedules and on-call work. A CO described his work hours below:

*“…my shift is eight to four regularly but other than that it’s either eight to ten, so it’s 14 hours… And I do swaps also, so it could be a 14-hour day, then a 16-hour day followed by another 14-hour day. Since I’m, I have low seniority, I’ll get mandated more often, so it kinda jumps around. Sometimes I’ll be here ‘til four or, I’m sorry, ‘til seven, eight, here ‘til ten, here ‘til twelve.”* (Male correctional officer)

Manufacturing workers reported different job descriptions - technician, driver, operator, process engineer, environmental team leader, logistics coordinator, security officer and gatekeeper. All workers had varying and changing shifts (52 different shifts occur across the company), either 8, 12 or 24 hours a day, but typically 12-hour shifts. Schedules could either be permanent or rotating, with a lot of mandatory overtime. Below are examples of worker-described typical work hours and schedules:

“*I worked permanent third shift the first year I was here, and the only little part they forgot to tell me that there was no days off. I worked every day for almost a year. And if you wanted a day off, you had to ask for it…*” (Male manufacturing worker)

“*I worked rotating. I’d work first shift, second shift on the weekend, second shift through, third shift on the weekends through, then I’d come back on the second shift on a weekend and go down to days for the next week. And do that every week.*” (Male manufacturing worker)

Themes, sub-themes and sub-theme frequencies for worker-identified barriers of work hours and schedules to health and well-being are summarized in Table 3. Each theme and sub-theme with quotation examples are detailed below:

**Table 3:** Barriers of work hours and schedule to health and well-being

Themes	Sub-themes	Frequency (n)
Well-being behavior impacts	Strained family connections	83
	Poor eating	76
	Poor sleep	59
	Poor social and/or community connections	34
	Lack of exercise	16
Physical health impacts	Exhaustion	29
	Weight gain	5
Extended exposures from work	Increased hypervigilance, stress/anxiety	17
	Increased accidents at work/mistakes on the job	6

### Theme 1: Well-being behavior impacts

Workers reported numerous ways that their extended time at work affected the behaviors used to maintain their well-being. These pertained to physical and mental health, and family, social, and community connections. (We present sub-themes in order of descending frequency).

#### Sub-theme 1.1 Strained family connections (*n*=83)

Workers reported strained family relationships as a result of missing important and routine events with family due to long work hours, irregular schedules and shift work.

*“You miss out on so much.... Your family events...holidays...with your family...like this year because of working weekends, I barely saw any of my daughter’s soccer games …– I mean they give you a lot of time, but then they restrict how you use it. You feel like – you're a bad mom. You're a bad friend. You're a bad family member because you can't be there for a lot of people’s things.”* (Female correctional officer)

*“My wife told me she’d divorce me the first year I was here if I didn’t get off that schedule. I was workin’ every single day.”* (Male manufacturing worker)

#### Sub-theme 1.2 Poor eating (*n*=76)

Workers indicated changing and irregular eating patterns as well as unhealthy eating due to lack of access to healthy food from working extended/irregular hours and night work.

*“...and if you are at work, you’re eating stuff you shouldn’t be eating... Junk food or somethin’ that you shouldn’t ‘cause your body’s naturally asleep in those hours and now you’re puttin’ in some junk that you don’t need….Cause what else is there to eat? What else is there to eat at 12:00 or 1:00 in the morning?”* (Male correctional supervisor)

*“You can't do a lot of things you usually do in the summertime, so that’s kind of tough but as soon as the warm weather hits, a lot of guys try and do healthy stuff, start eating right again, and we see a lot of guys on their bikes, walking. I try to do more healthy stuff in the summertime.”* (Male transportation maintainer)

#### Sub-theme 1.3 Poor sleep (*n*=59)

Workers reported difficulties with changing sleeping patterns and poor sleep quantity and quality due to long and irregular work hours and night work.

*“... and then you’re losing out on sleep and everybody goes, oh, just sleep whenever, and that doesn’t really work like that, so you’re tryin’ to sleep two hours in the morning when you get home and then a couple hours in the afternoon before you go back to work, and it just doesn’t, doesn’t end well.”* (Male manufacturing worker)

*“… I changed my whole regimen around, so it was okay. I changed my sleep pattern around, so my 3:00 in the morning would be like somebody’s 7:00 at night…”* (Male correctional supervisor)

*“…I don’t think – especially on a double, you can’t – or even workin’ second shift. Like I said, even when I get home and – when I worked second, which was probably the busiest shift in the facility, second shift, you get off, you’re so wired, you can’t sleep...”* (Male correctional supervisor)

#### Sub-theme 1.4 Poor social and/or community connections (*n*=34)

Workers reported missing everyday social activities, opportunities to volunteer in the community, and also educational opportunities due to long work hours, irregular schedules and shift work.

*“My wife’s friends call me a unicorn. I’m the mythical creature she talks about but none of’m have ever seen me. I’m always here (at work) when they’re doin’ things.”* (Male correctional officer)

*“I always wanted to do Big Brother/Big Sister...But never did it because your schedule changes a lotta times, so then you end up – you take the individual to help them out, but if you’re never available to help them out,... for them to count on you or they – it’s always a younger-type individual. They’re not gonna be able to sit there and figure out five days on and three days off, five days on. It’s after school today or whatever.”* (Female correctional supervisor)

*“Yeah, that rotating schedule is, I told’m, I turned down job offers here because of the rotating schedule because I can’t do that. With college, you can’t plan on goin’ to class if you’re gonna miss out on half the class.”* (Male manufacturing worker)

#### Sub-theme 1.5 Lack of exercise (*n*=16)

Workers reported that long work hours leave them with no time to exercise.

*“…So no physical activity ‘cause you’re sitting in a truck...for 20 hours straight…”* (Male transportation maintainer)

*“For me,…I do work a lotta double shifts. That’s by choice for family reasons, so as for the aspects for the health is, I don’t get to the gym as much as I choose to. I’d like to go to the gym every day but because I work so many double shifts… Because when you’re workin’ 16-hour days, it’s kinda hard to eke out 45 minutes to an hour to get to the days – to get to the gym, so it’s either what are you gonna do? You gonna make money or you gonna get buff. Like it’s…it’s hard to balance’m both, especially if you’re, excuse me, if you’re a five-day-a-week type a dude.”* (Male correctional officer)

### Theme 2: Physical health impacts

Workers identified several ways that long and irregular work hours and schedules affect their physical health and well-being including exhaustion and weight gain.

#### Sub-theme 2.1 Exhaustion (*n*=29)

Workers reported that long work hours resulted in physical exhaustion.

*“I mean it's just general exhaustion. So it pretty much hampers your ability to do anything...When you get home, you just go right to sleep...”* (Male transportation maintainer)

*“Yeah. I guess, yeah, winter ‘cause you get – you work 40-something hours straight and you start to get tired and...It takes a toll on your body, you do it year after year after year.”* (Male transportation maintainer)

*“...Like I said, I got outta work Monday, go home, go right to bed, just almost, just don’t feel like doin’ any, just too tired to do anything. It just messes up your outside…”* (Male manufacturing worker)

#### Sub-theme 2.2 Weight gain (*n*=5)

Workers reported weight gain as a result of poor eating and lack of exercise due to irregular schedules and night work.

*“It's a strenuous time, wintertime… and like this time of year, I work out a lot. I jog. I lift weights. I do a lot. I stay active. But that ends once winter…and then you're not eating the healthiest food. It's hard to eat healthy. They say well you could choose to eat something healthy, but once you get – you know you're working like this, when you hungry, you're hungry, you gotta eat. And I find myself, I pack on at least about 20 pounds every winter...”* (Male transportation maintainer)

*“Oh, the negative way is like he said, gainin’ weight. I got high blood pressure, so durin’ the wintertime, I don’t have any type of – since – actually, the truth is, when I got to the State, that’s when my high blood pressure went up ‘cause I wasn’t – not because of the job. It’s because I don’t – the physical activity of the job like night – most days, I’m in the truck sittin’ on a highway. I’m not doin’ a lot of physical like active work like I was doin’ before…, so physical activity was a lot more than it was prior to me comin’ here. So with the wintertime, the three or four months in the winter and then in the summertime, runnin’ machinery or sittin’ on machinery or sittin’ in the trucks, I don’t have the activity no more and I don’t work out after work or go after work, so that’s what affects me prior to bein’ here.”* (Male transportation maintainer)

### Theme 3: Extended exposures at work

Workers identified ways that their health and well-being were affected by extended exposures to work hazards, including increased hypervigilance, stress and anxiety and increased chances of accidents or mistakes on the job.

#### Sub-theme 3.1 Increased hypervigilance, stress/anxiety (*n*=17)

Due to extended work hours in a dangerous and/or stressful work environment, workers reported increased psychological symptoms of hypervigilance, stress and anxiety. The majority of comments (*n*=13) in this category were from workers in corrections with the remainder (*n*=4) from transportation workers.

*“… so here…everything’s supposed to be secured, so me walkin’ down any hallway constantly just touching doorknobs to make sure…the doors are secured. You get home. Now, here I am, it’s late night, it’s dark out, you’re puttin’ the kids to bed, you lock up the house. Right? You go sit down, you’re tryin’ to relax, unwind, maybe watch TV, read a book, and you’re like… is that door unlocked? Go get up, just to go check the door to make sure it was secured.”* (Male correctional officer)

*“Oh, yeah. Especially if I've done a lot of doubles…and I'm irritable. Oh, I will go off the handle.”* (Male correctional officer)

*“It can just be stressful sometimes. It's like in the wintertime like you're here so long, you're looking at the road, you can get tunnel vision, so it's mentally, you know what I mean, sometimes more than physically stressful… ‘Cause you're here so long… you just want to go home. ”* (Male transportation maintainer)

#### Sub-theme 3.2 Increased accidents at work/mistakes on the job (*n*=6)

Due to physically demanding work over long hours, workers reported sustaining injuries or making mistakes on the job. Of the 6 comments, 5 came from corrections workers and 1 from a transportation maintainer.

*“… I’ve seen people now that – normal guys,…if they work one shift, they’re good. But…I see how many hours they’re workin’. There’s a correlation. The more you work, the more chances you make mistakes.”* (Male correctional officer)

*“…sometime driving too many hours, and we have to be safe, no accidents…”* (Male transportation maintainer)

*“…I work a lot of overtime… I’ve run to a couple of codes, injured my knee, tore up my rotator cuff, my bicep, my hand’s still not, some days it’s real rough…”* (Female correctional officer)

Despite the long and irregular hours these workers spend at work, focus group participants identified some aspects of their work hours and schedules that gave them schedule control and were benefits to worker health and well-being. Six sub-themes emerged from the main theme: Well-being behavior impacts (Table 4). Sub-theme frequencies, sub-themes and the main theme for worker-identified benefits of work hours and schedules to health and well-being are summarized in Table 4. Each theme and sub-theme with quotation examples are detailed below:

**Table 4:** Benefits of work hours and schedule to health and well-being

Main theme	Sub-themes	Frequency (n)
Well-being behavior impacts	Financial benefits	31
	Alone time	28
	More sleep	8
	Social connections at work	7
	Time for family connections	7
	Less/reduced stress	4

### Theme 1: Well-being behavior impacts

Workers identified some ways in which their long and irregular schedules benefited their ability to maintain well-being behaviors including financial benefits, time for themselves, getting more sleep, work social connections, time for family connections and reducing stress levels.

#### Sub-theme 1.1 Financial benefits (*n*= 31)

Workers reported earning more money as a result of longer hours spent at work due to overtime and differentials. They spent the extra money earned on behaviors which helped to maintain their health and well-being such as taking vacation, paying bills, paying for children’s education and buying things.

*“...it’s rough on our health, but it pays well, so you could accumulate quite a few toys [laughter] if you want to.”* (Male transportation maintainer)

*“The only benefit I can foresee is … with working long hours in the winter making extra money… the summer, using that money maybe to do a long vacation and something that we normally wouldn't be able to afford and something nice or cruisin’ around in a nice car or.. Puttin’ kids through college.”* (Male transportation maintainer)

*“…Yeah, the money [laughter]….That’s the one thin’ that I can say. I thank god for it. I don’t complain about my job, of course, nobody’s job is perfect, but I feel like this is probably one of the only jobs that you can have and be able to say I wanna go on vacation, what are we, in August? Okay, I wanna go on vacation end of September. You can be able to work what you wanna work so that you have that extra money. A lotta people don’t have that luxury. If you work a nine to five job Monday through Friday, you’ll be savin’ up for a lifetime for a vacation…”* (Female correctional officer)

#### Sub-theme 1.2 Alone time (*n*=28)

Workers reported some free and alone time from their shifts or getting days off after their long and irregular schedules which benefited their health and well-being.

*“…I had more free time on second and third shift… I’d use it for relaxation or goin’ out and playin’ golf or somethin’ like that because workin’ first shift, there’s really no option and the weekend, it’s busy. Durin’ the week, if you’re off durin’ the week durin’ the day, it’s – you can go and there’s nobody else out.”* (Male correctional supervisor)

*“Well, for me, it’s getting off at one o’clock is really beneficial. There’s a lotta stuff that I – paying bills or running errands or that kind of, some yard work. Take advantage of that time to have by myself before the family comes home…”* (Male manufacturing worker)

#### Sub-theme 1.3 Sleep (*n*=8)

Due to longer hours, differentials and irregular shifts (such as night shift), workers were able to work out their schedules and shifts to catch up on sleep and maintain their sleep patterns.

*“…second shift was great. You could sleep late in the day…”* (Male correctional supervisor)

*“Whoever relieves you. So I’ve always said it to the younger guys, I don’t care if I was you, I would tell the guy he could have the days and I’d take the nights or he can get the nights and you take the days, but don’t rotate. I said, ‘cause you ain’t got no life...Then your sleep patterns stay the same.”* (Male manufacturing worker)

#### Sub-theme 1.4 Social connections at work (*n*=7)

Workers reported building social connections with coworkers due to the long hours they spent together at work.

*“… you kinda build a family with the people that you work with because sometimes you’re at work when you’re at home.”* (Male correctional supervisor)

*“… And you get to know these people. Like this is my sister. These are my brothers right here. Like I care about them…that’s my big aunt right there, so me, the type of person that I am, I do take that personally…You know what I’m saying? Because I got love for all these people…”* (Male correctional officer)

#### Sub-theme 1.5 Time for family connections (*n*=7)

Workers reported having some time to spend with family on some shifts and schedules.

*“Eight to four Monday through Friday, you know that …you h-, you don’t have to answer the phone during the summer, and you can enjoy your weekends with your family...”* (Male transportation worker)

*“...after the long winter and doing more activities outside... You spend more time – well you get a little rest first. You take a couple of week and get rested, and then once the weather turns nicer, that’s spending time with family and doing more stuff outside…”* (Male transportation worker)

*“So like you said, me, my kids were all C-section, so it’s a planned date birth...So I know on this day we’re goin’ in, so I take my three (week) baby leave. Then I swap out two, so that’s one week off. Then the next week I swap all five of those off, now I have 19 days off to spend with my wife and my newborn without burnin’ all my time up...To be home to help the wife out while she heals and stuff like that.”* (Male correctional officer)

#### Sub-theme 1.6 Reduced stress (*n*=4)

Workers recounted some ways that aspects of their long work hours and schedules reduce their stress levels, due to reduced exposure from work, or the benefits they get from working long hours such as the extra money which reduces stress.

*“Well it's a big improvement after April 30th…Even though we get called in, but it's nothing like the wintertime. So a lot of times that we able to do the things that we need to do. It's not as demanding.”* (Male transportation maintainer)

*“Yep. I look at it, I tell you what my job’s all about. It’s one of the low, it is the lowest paying job in this mill but the quality of life…the quality of life, I have opportunities to get those $33 an hour jobs and I’m goin’ why? Then you get into the rotating shifts, you get nights, days, week, no more weekends. This, I look at, I gotta good benefit package and there’s hardly any money, but it’s easy to come in for eight hours a day and go home. I watch all these other guys goin’, and they struggle, and I’m goin’ holy cow. I don’t want that.”* (Male manufacturing worker)

*“…But you pay your bills, you feel better...You're not stressing out.”* (Male transportation maintainer)

## Discussion

Full-time workers with NSWSs reported numerous ways in which their work schedules impacted personal and family well-being. Consistent with the literature, workers reported NSWS affects across health-related outcomes in the domains of physiological, psychological and health behaviors, personal/family-related outcomes as well as work-related outcomes [5]. The work schedule impacts reported by workers were supported across three themes – well-being behaviors, physical health, and extended exposures at work. Impaired social connections through strained family connections and poor social and community connections were most frequently noted as being impacted by work schedules. Along with increased hypervigilance, stress, and/or anxiety, these factors have the potential to precipitate mental health effects. Direct impacts on physical health were noted through exhaustion and weight gain along with indirect physical health impacts through health behaviors including poor eating, poor sleep, and lack of exercise. To a small extent, workers reported work outcomes including increased accidents at work.

Despite different industry sectors and job titles, the workers in this study were similar in that all were full-time employees who experienced NSWSs. Extended work hours through overtime was characteristic for workers in all three sectors, as was irregularity and little notice of extended work. However, workers reported variations in other characteristics and patterns of the work. The transportation maintainers consistently worked first shift and experienced seasonal variation in work shifts due to storm work. While the correctional officers and correctional supervisors worked rotating shifts with mandatory overtime consistent throughout the year. In addition to mandatory overtime in the form of double shifts, correctional officers also reported swapping shifts with colleagues in order to get some time off of work which contributes to increased schedule length and irregularity. The manufacturing employees worked varied shift configurations, based on the production line and machinery that they were assigned with overtime being driven by production schedules.

### Social connections

Consistent with the proposed mechanisms by which shift work impacts health [6], we observed social disruption as a major threat to worker and family well-being. Social disruption was expressed by workers in all industries. Strained family connections was the most frequent well-being impact cited by workers. Related, poor social and community connections were also cited as a barrier to well-being. Research supports how NSWSs affect the balance between work and non-work domains including social participation [8]. Working and living patterns that are asynchronous with the community rhythms of family, community and recreational activity can precipitate work-life conflict, especially in women where work stress in combination with high family responsibilities leads to increase in psychological distress [18]. While the majority of the current population was male, the majority (81%) also had child and/or adult dependents. Yet, within the current population, the effects of work schedule on social consequences were also reported by single workers who reported missing engagement with friends. In fact, workers noted the difficulty of missing out on important as well as routine family and social events ranging from weddings to children’s sports events, due to work schedule challenges.

### Healthy eating, exercise and weight

The next most frequently mentioned barrier of schedules to well-being was in the category of physical health and health behaviors including healthy eating and exercise. This qualitative finding was in line with previous quantitative research among corrections officers. In a cross-sectional survey of 157 correctional supervisors, we found a high prevalence of obesity with 38% categorized as overweight and 51% obese [19]. Furthermore, health supporting behaviors in the population were likewise low with 27% reporting often or always meeting recommended fruit intake and 42% often or always meeting physical activity guidelines [19]. Worker perspectives confirmed the role of work schedules in influencing poor health and health behaviors among correctional workers. Similar perspectives were echoed among transportation maintainers where weight gain increased over the winter months due to long hours of sitting (driving), lack of physical activity and poor eating choices and patterns. The effect of NSWSs on poor eating behaviors includes irregular eating behaviors, heightened food cravings, and increased food and fat intake [5, 20]. Poor eating habits in combination with circadian disruption [21] and sleep debt [22] may contribute to the increased obesity associated with shift work [23, 24]. The lack of exercise among workers with NSWSs may also contribute to obesity risk. Night work has been shown to be associated with less exercise in assisted living caregivers [25], although not in nurses [26]. The link between NSWSs and exercise is not fully explored.

### Sleep and fatigue

Poor sleep and exhaustion were noted by workers. The role of sleep and exhaustion resulting from lack of recovery is well explored in our understanding of work schedules and health [5]. While recognized as being impacted by work schedules, sleep and fatigue were not as frequently mentioned as other factors. In a qualitative study of transit worker stress, workers reported putting family before sleep [12]. In fact, night shift workers often choose the shift to better balance family responsibilities, despite the trade-off in terms of sleep [27].

### Increased exposures at work

Workers, especially those in corrections, reported increased exposures at work. Importantly, the workplace exposures vary by sector. Transportation workers have physically demanding jobs with numerous physical and over-exertion hazards. Whereas corrections workers may have increased exposure to psychosocial hazards from extended exposures to dangers and stressors at work and may be at increased risk for hypervigilance, stress and anxiety. While fatigue and its related consequences such as increased risk for accidents is a well-known consequence of extended exposures at work [28], it is important to recognize that additional risks may be relevant based on job-hazards as well as worker characteristics [29].

### Financial benefits

Across all work groups, financial rewards were cited as the most important benefits of their work schedules. Workers reported a wide range of financial expenditures ranging from household items, to children’s college costs, to family vacations, to luxury items such as cars. From a worker’s perspective, incentives of US workers for long work hours, and more broadly NSWSs, are largely financial with increased pay and often times advancement by accepting the employers’ overtime or work shift requests. Corrections workers in the state where the data was collected report a common belief within the organization that health must be sacrificed to maximize present and future income [30]. Overtime pay provides a substantial boost to current income and enables workers and their families to attain a very high standard of living, which they become accustomed to. Moreover, the retirement policy enables workers to retire after 20 years of service, with their payout being based on the highest 3 years of earnings. Thus there is a great deal of pressure to maximize earnings through overtime work, especially late in one’s career. However, it is important to remember that NSWSs are a benefit to the employer as well. In fact, long work hours, can be a result of increased production demands where employers seek to minimize costs through overtime, rather than paying employee benefit costs that are incurred with hiring additional employees [29].

### Strengths and limitations of qualitative study design

Through qualitative research methods, we identified a breadth of well-being impacts due to work hours and schedules. From a worker perspective, social connections were deeply affected by NSWS. While prior work suggests that working on weekends impacts worker well-being [8], based on worker perspectives indicate that missing important events, which may or may not be on weekends, has impacts on worker and family well-being.

As with many qualitative research studies, there are limitations to the generalizability of our results to other populations. Importantly, we engaged full-time workers with long and irregular work hours. Their experiences are likely different from workers with precarious schedules who may also experience irregular and extended hours without the benefit of stable full-time work or benefits. The majority of focus group participants were men, which is a reflection of the male-dominated transportation, corrections and manufacturing industries. The threats of work schedules to the well-being of women are likely different as women face additional responsibilities inside the home. Likewise while workers were recruited based on their self-reported experience of NSWS, the majority of workers who were interviewed worked first shift and more detailed information about NSWS shift characteristics were not collected. Workers who experienced the most deleterious effects of work schedules may leave the workforce and their perspective is not likely captured in this cross-sectional study.

### Future research directions

The worker perspectives from these focus groups informed the development of survey items that captured the full breadth of worker impacts as well as schedule characteristics. The worker perspectives made evident that a broader definition of well-being, which, in addition to encompassing physical and mental health, should also include social connections and financial security, is needed when assessing the impacts and benefits of NSWSs. This is consistent with the *Total Worker Health* approach that recognizes worker well-being is influenced by a set of expanded domains which, in addition to considering the workplace physical environment and culture, also considers workers health status, evaluation of work and home experience, community and societal engagement, and satisfaction [2]. Consistent with the NIOSH *Total Worker Health* framework, work schedules had impacts across health status, social relationships, and within the workplace itself [2]. In fact, recognizing and addressing work organization impacts, which includes work schedule characteristics, has been suggested as a defining element of *Total Worker Health *approaches [31].

With respect to work organization, future interventions (including work schedule changes), should address prevention across the spectrum from primary to tertiary prevention. For example, in a multi-level approach for managing workers’ sleep-related fatigue, primary prevention may include providing sleep opportunity through limiting hours of service and adding rest breaks. While tertiary prevention may include reducing fatigue-related incidents [32]. Primary through tertiary prevention approaches may also be relevant for mental and physical health and health behavior consequences. A multi-level perspective should also be considered with respect to the target of interventions; in addition to interventions on the individual level, it is also imperative to intervene at the job/task level, the employer/organization level and the legislative/policy level [33]. For example, interventions to address the increased hypervigilance and stress reported by workers with NSWS may include primary prevention at the organizational level that adds more schedule control and increased staffing to reduce long work hours while also providing tertiary prevention at the individual level by educating workers about stress and hypervigilance and providing best practices for stress reduction.

## Conclusion

This qualitative study was part of the larger WorkTime project focused on understanding the effects of nonstandard work schedules on worker and family well-being. Worker perspectives highlighted social disruptions as a salient hardship of NSWSs in this full-time, blue-collar work force.

## Data Availability

Data sharing is not applicable to this article as no datasets were generated or analyzed during the current study*.*
